# The Hemodynamic Changes Induced by Lung Recruitment Maneuver to Predict Fluid Responsiveness in Children during One Lung Ventilation—A Prospective Observational Study

**DOI:** 10.3390/children11060649

**Published:** 2024-05-27

**Authors:** Ting Liu, Pan He, Jie Hu, Yanting Wang, Yang Shen, Zhezhe Peng, Ying Sun

**Affiliations:** Department of Anesthesiology, Shanghai Children’s Medical Center, School of Medicine and National Children’s Medical Center, Shanghai Jiao Tong University, Shanghai 200127, China; tinga277@163.com (T.L.); hepan@scmc.com.cn (P.H.); hujie@scmc.com.cn (J.H.); wangyanting@scmc.com.cn (Y.W.); shenyang@scmc.com.cn (Y.S.)

**Keywords:** one-lung ventilation, lateral thoracotomy, cardiac surgery, child, postoperative pulmonary complications

## Abstract

Background: The prediction of fluid responsiveness in critical patients helps clinicians in decision making to avoid either under- or overloading of fluid. This study was designed to determine whether lung recruitment maneuver (LRM) would have an effect on the predictability of fluid responsiveness by the changes of hemodynamic parameters in pediatric patients who were receiving lung-protective ventilation and one-lung ventilation (OLV). Methods: A total of 34 children, aged 1–6 years old, scheduled for heart surgeries via right thoracotomy were enrolled. Patients were anesthetized and OLV with lung-protection ventilation settings was established, and then, positioned on left lateral decubitus. LRM and volume expansion (VE) were performed in sequence. Heart rate (HR), systolic arterial pressure (SAP), mean arterial pressure (MAP) diastolic arterial pressure (DAP), stroke volume (SV), stroke volume variation (SVV), and pulse pressure variation (PPV) were recorded via an A-line based monitor system at the following time points: before and after LRM (T1 and T2) and before and after VE (T3 and T4). An increase in stroke volume (SV) or mean arterial pressure (MAP) of ≥10% following fluid loading identified fluid responders. The predictability of fluid responsiveness by the changes of SV (ΔSV_LRM_) and MAP (ΔMAP_LRM_) after LRM and VE were statistically evaluated by receiver operating characteristic curves [area under the curves (AUC)]. Results: SVs in all patients were significantly decreased after LRM (*p* < 0.01) and then, increased and returned to baseline after VE (*p* < 0.01). In total, 16 out of 34 patients who were fluid responders had significantly lower SV after LRM compared to that in fluid non-responders. The area under the receiver operating characteristic curves for ΔSV_LRM_ was 0.828 (95% confidence interval [CI], 0.660 to 0.935; *p* < 0.001) and it indicated that ΔSV_LRM_ was able to predict the fluid responsiveness of pediatric patients. MAPs in all patients were also decreased significantly after LRM, and 12 of them fell into the category of fluid responders after VE. Statistically, ΔMAP_LRM_ did not predict fluid responsiveness when LRM was considered as an influential factor (*p =* 0.07). Conclusions: ΔSV_LRM_, but not ΔMAP_LRM_, showed great reliability in the prediction of the fluid responsiveness following VE in children during one-lung ventilation with lung-protective settings. Trial registration: ChiCTR2300070690.

## 1. Introduction

One lung ventilation is commonly used in thoracic procedures [1], especially in children undergoing surgeries for congenital heart diseases. Proper fluid management is crucial during OLV to avoid pulmonary edema, especially considering the narrow fluid tolerance in pediatric patients with congenital heart disease (CHD). However, inadequate fluid replacement could compromise mean arterial blood pressure (MAP) and cardiac output (CO), and subsequently, diminish tissue perfusion and oxygenation [2].

Fluid responsiveness in patients can be assessed by static and dynamic predictors. A consensus has been reached that the reliability of static indices is too poor to distinguish the differences in fluid responsiveness in both adults and pediatric patients. Several widely investigated dynamic indices such as pulse pressure variation (PPV), stroke volume variation (SVV) and pleth variability index (PVI) have shown better results in predicting the response to fluid administration in mechanically ventilated pediatric patients [3,4,5]. However, respiratory variation in aortic blood flow peak velocity (∆Vpeak) has been proven to be a reliable dynamic parameter for predicting responses to fluid loading in children but its reliance on echocardiography limits practicality.

Studies have shown that the detection of fluid responsiveness in mechanically ventilated patients require a minimal tidal volume of 8 mL/kg and driving airway pressure of 20 cmH_2_O [6,7,8,9]. During OLV, the lung-protective ventilation settings with lower tidal volume (6 mL/kg) and higher positive end-expiratory pressure (PEEP) are recommended. Theoretically, the reliability or sensitivity of dynamic predictors of fluid responsiveness during OLV might be compromised when a smaller tidal volume and lower airway driving pressure are delivered [6,7,8,9].

High oxygen concentration, including pure oxygen has been shown in speeding lung collapse. LRM are routinely used to reopen collapsed alveoli during OLV in children undergoing heart surgeries. However, these maneuvers may lead to transient decreases in stroke volume and blood pressure, mimicking a hypovolemic state. Monitoring dynamic preload parameters may improve sensitivity in detecting fluid responsiveness [9,10]. A previous study proved that the decrease in SV induced by LRM could predict fluid responsiveness in the operation room [11]. Recently, Kimura et al. found that the decrease in SV and BP prompted by LRM during OLV showed better predictability of fluid responsiveness in adults [10]. Further studies are needed to assess the impact of these maneuvers on fluid responsiveness in pediatric patients.

We carried out this study to determine whether LRM-induced changes of SV and BP might discriminate fluid responders from fluid non-responders in pediatric patients during OLV and lung-protection ventilation.

## 2. Methods

This prospective observational study was carried out over the period from April to June 2023. It was approved by the Institutional Review Board of Shanghai Children’s Medical Centre on 31 March 2023 (SCMCIRB-K2023046-1) and was registered at Chinese Clinical Trial Registry (ChiCTR2300070690). Written informed consent was obtained from the parents or legal guardians of children the day before surgery.

### 2.1. Study Population

Children, aged 1 to 6 years, ASA Physical Status (ASA-PS) 2 or 3, and scheduled for surgical closure of ventricular septal defect (VSD) or atrial septal defect (ASD) through a right lateral thoracotomy with cardiopulmonary bypass (CPB), were enrolled in this study. Patients having preoperative arrhythmia and ventricular dysfunction (ejection fraction < 50%), or with high pulmonary hypertension and respiratory system diseases were excluded. Each participant adhered to the same fasting duration before elective surgery.

### 2.2. Anesthesia and Establishment of OLV

Standard monitoring, including ECG, BP, pulse oxygen saturation (SpO_2_), and body temperature was incorporated. With 5 L·min^−1^ oxygen flow, 100% inhaled oxygen concentration (FiO_2_), anesthesia was induced with intravenous (IV) midazolam 0.1 mg·kg^−1^, etomidate 0.2–0.3 mg·kg^−1^, sufentanil 2 μg·kg^−1^, and rocuronium 0.6 mg·kg^−1^, and was maintained with continuous IV infusion of 4.0–6.0 mg·kg^−1^·h^−1^ propofol, 2.0–2.5 μg·kg^−1^·h^−1^ sufentanil, and 0.6 mg·kg^−1^·h^−1^ rocuronium. Tracheal intubation was performed and then a bronchial blocker was placed into the right bronchus under fiberoptic bronchoscopic guidance. Its position was confirmed by bilateral auscultation. The right lung was deflated by removing air through a suction port. Patients were placed to the left lateral decubitus and mechanical OLV with lung-protection ventilation was maintained in pressure-controlled volume-guaranteed (PCV–VG) mode: tidal volume 6 mL·kg^−1^, positive end-expiratory pressure 6 cmH_2_O, respiratory rate was adjusted to maintain end-tidal partial pressure of CO_2_ between 40 and 50 mmHg. Mean pressure measured during OLV was 17–20 cmH_2_O, which was lower than the preset pressure of operating LRM. A fractional inspired oxygen tension (FiO_2_) of 0.5 and I: E (inspiration: expiration ratio) was 1:2.

### 2.3. Hemodynamic Monitoring

A radial A-line (dependent arm) was placed and heart rate (HR), systolic arterial pressure (SAP), diastolic arterial pressure (DAP), mean arterial pressure (MAP), stroke volume (SV), pulse pressure variation (PPV), and stroke volume variation (SVV) were continuously recorded throughout the investigational period with a LiDCO System (LiDCO Ltd., Cambridge, UK). This system provides beat-to-beat advanced hemodynamic monitoring by using an already existing arterial line to provide decision-making information in the ICU and operating room.

### 2.4. Lung Recruitment Maneuver (LRM) and Volume Expansion (VE)

LRM was conducted by an independent researcher who was totally blinded to any hemodynamic data registration. After patients were appropriately positioned, LRM was performed through a cuffed tracheal tube to the left lung (dependent) with a continuous inspiratory pressure of 25 cmH_2_O for 20 s. 5 min at the completion of LRM; VE was carried out with 10 mL·kg^−1^ of Ringer’s solution over 10 min. The hemodynamic variables were collected immediately before and after LRM (T1 and T2), and immediately before and after VE (T3 and T4) by another investigator. The trends of hemodynamic parameters induced by LRM or VE (to reflex the fluid responsiveness) were calculated as follows:

(1) $$\Delta {\mathrm{SV}}_{\mathrm{LRM}}=\;\frac{SV\;at\;the\;end\;of\;recruitment\;maneuver-SV\;before\;recruitment\;maneuver}{SV\;before\;recruitment\;maneuver}\times 100$$

(2) $$\Delta {\mathrm{SV}}_{\mathrm{VE}}=\;\frac{SV\;after\;volume\;expansion-SV\;before\;volume\;expansion}{SV\;before\;volume\;expansion}\times 100$$

(3) $$\Delta {\mathrm{MAP}}_{\mathrm{LRM}}=\;\frac{MAP\;at\;the\;end\;of\;recruitment\;maneuver-MAP\;before\;recruitment\;maneuver}{MAP\;before\;recruitment\;maneuver}\times 100$$

(4) $$\Delta {\mathrm{MAP}}_{\mathrm{VE}}=\;\frac{MAP\;after\;volume\;expansion-MAP\;before\;volume\;expansion}{MAP\;before\;volume\;expansion}\times 100$$

### 2.5. Statistical Analysis

The primary end point of this study was the fluid responsiveness predictability indicated by LRM-induced ΔSV (ΔSV_LRM_) and LRM-induced ΔMAP (ΔMAP_LRM_). The predictability of fluid responsiveness was analyzed by calculating the area under the curve (AUC) of the receiver operating characteristic curves (ROCs).

Aky et al. reported that the AUC of the ROC of ΔSV_LRM_ to predict fluid responsiveness in adult patients was 0.84, and we assumed that the AUC for ΔSV_LRM_ would be 0.8. The sample size was calculated by using MedCalc software, version 19.8 (Ostend, Belgium). The ratio of patients who did respond (fluid responder) vs. those who did not respond to fluid expansion (non-responder) assessed by the changes of SV and MAP following VE were assumed to be 1:2 according to the results of the previous study [12]. With α error of 0.05, power of 0.8, and the null hypothesis of an AUC of 0.5 [13], and a potential dropout of 10%, 34 patients were finally enrolled in this study.

The fluid responsiveness was defined as ≥10% increase in SV or MAP after VE [10,11]. The Kolmogorov–Smirnov test was used to test the assumption of the normal distribution and data were expressed as mean ± standard deviation (SD) or the number (percentage, %). The hemodynamic variables at each time point, before and after LRM (T1 versus T2) and before and after VE (T3 versus T4), were compared using the nonparametric Wilcoxon rank sum test and Student’s paired *t* test. ROCs were painted for ΔSV_LRM_, ΔMAP_LRM_, SVV1 (SVV at T1), SVV3 (SVV at T3), PPV1 (PPV at T1), and PPV3 (PPV at T3). The AUC of the variables was compared using the method of DeLong et al. [14].

Furthermore, the gray zone approach was used to recognize inconclusive ranges of ΔSV_LRM_ and ΔMAP_LRM_ (whether the subjects were fluid responders or fluid non-responders). First, bootstrap resampling of 1000 estimated thresholds was performed to identify the best cutoff point and 95% confidence interval (CI). Secondly, 3 response classes (negative, inconclusive, and positive) were used to calculate the gray zone of ΔSV_LRM_, and ΔMAP_LRM_; the range of inconclusive responses were cutoff values with a sensitivity and a specificity of <90% [9,10]. The larger range calculated between the two steps was the gray zone [13]. A *p* value < 0.05 was considered to be statistically significant. Statistical analysis was performed with Medcalc software, version 14.8.1 (Mariakerke, Belgium) and SPSS software, version 26 (IBM, NewYork, NY, USA).

## 3. Results

A Total of 34 patients were enrolled in this study and their basic demographic data are described in Table 1. Table 2 shows the recorded hemodynamic parameters at time points of T1, T2, T3 and T4, and it shows that SV decreased sharply in all study participants at the end of LRM (*p* < 0.01) and then, increased significantly after VE (*p* < 0.01). MAP also lowered after LRM (*p* < 0.01); however, it did not increase significantly after VE when its value was compared to MAP before VE (*p =* 0.852).

**Figure 1:**
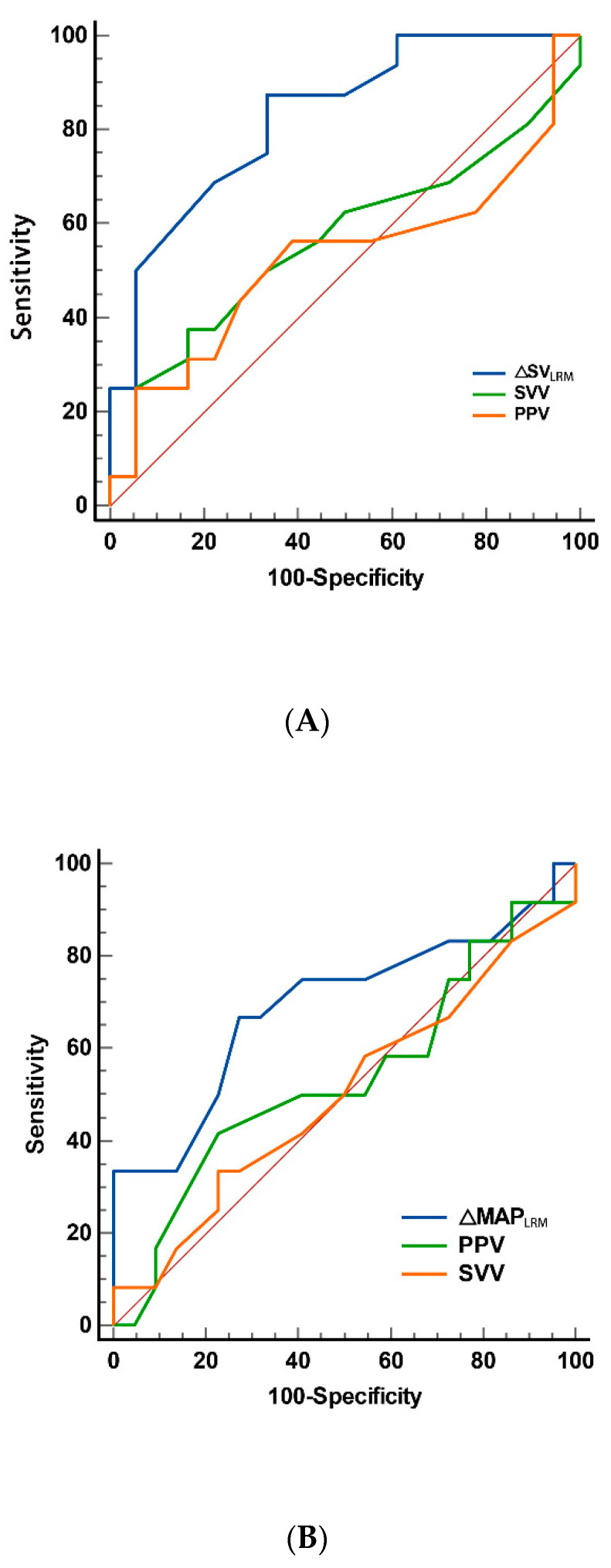
Receiver operating curves were generated from the changes of ΔSV_LRM_, SVV, and PPV induced by LRM (**A**), and the changes of ΔMAP_LRM_, SVV, and PPV induced by LRM (**B**). ΔSV_LRM_ showed the ability to predict the patients’ response to a loading dose of (10 mL·kg^−1^) Ringer’s solution over 10 min. Abbreviations: ΔMAP_LRM_, percent decrease in mean arterial pressure by recruitment maneuver; ΔSV_LRM_, percent decrease in stroke volume by recruitment maneuver; LRM, lung recruitment maneuver, continuous inspiratory pressure: 25 cmH_2_O for 20 s; MAP, mean arterial pressure; PPV, pulse pressure variation; SV, stroke volume; and SVV, stroke volume variation.

**Figure 2:**
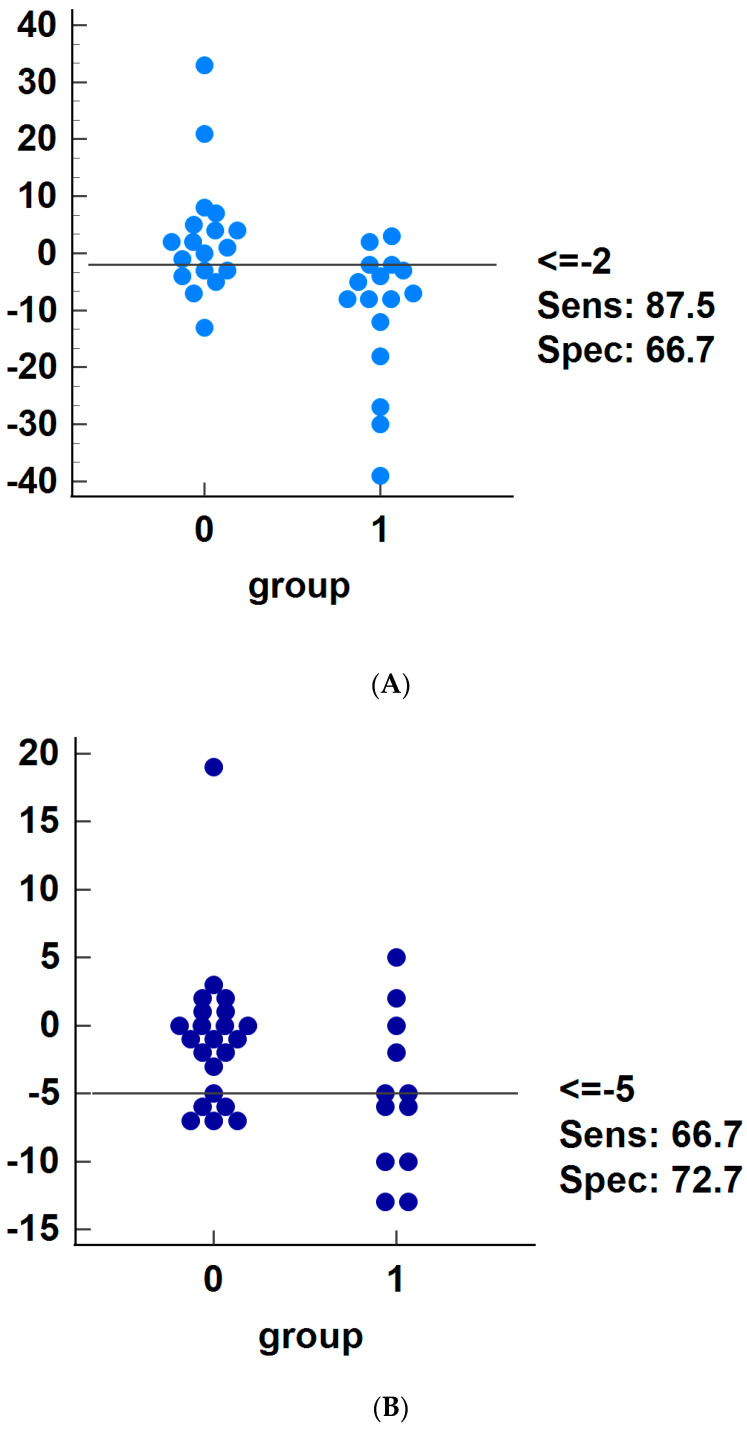
Interactive dot diagram. Individual values of changes in SV (%) induced by LRM (**A**) and of MAP (%) induced by LRM in responder (1) and non-responder patients (0) (**B**). Abbreviations: LRM, lung recruitment maneuver, continuous inspiratory pressure: 25 cmH_2_O for 20 s; MAP, mean arterial pressure; SV, stroke volume; Sens, sensitivity; and Spec, specificity.

Patients were categorized into fluid-responders if the increases of their SV or MAP were ≥10% after VE. Among 34 children, there were 16 SV-associated fluid responders and the calculated ΔSV_LRM_ (−10.50 ± 12.02%) in those patients was much lower than that in SV-non-responders (2.83 ± 10.41%) (*p =* 0.002). Moreover, ΔMAP_LRM_ exhibited a notably lower magnitude among fluid responders, with a reduction of −5.25% ± 5.74%, in stark contrast to the decrease of −0.91 ± 5.46% observed in the fluid non-responders (*p =* 0.037). Figure 1 exhibits the ROCs analysis to predict fluid responsiveness regarding the changes of SV (Figure 1A) and MAP (Figure 1B) before and after LRM. It can be seen from Table 3 that the AUC of ΔSV_LRM_, SVV, and PPV designated to discriminate SV-fluid responders were 0.828; 95% CI (0.660–0.935), *p* < 0.001, 0.611; 95% CI (0.429–0.773), *p =* 0.268, and 0.655; 95% CI (0.472–0.809), *p =* 0.118 respectively. The individual values of ΔSV_LRM_ before VE in responders and non-responders are shown in Figure 2A, and ΔSV_LRM_ accurately predicted fluid responsiveness in 47.06% of participants, with a cutoff value of −2%, a sensitivity of 87.50%, and a specificity of 66.67%. It can be seen from Table 4 that ΔMAP_LRM_, SVV, and PPV failed to discriminate fluid responders (ΔMAP_LRM_, AUC 0.693; 95% CI [0.512–0.839], *p =* 0.070, SVV, ROC_AUC_, 0.545; 95% CI [0.366–0.716], *p =* 0.682, PPV, AUC, 0.585; 95% CI [0.404–0.751], *p =* 0.414). The individual values of ΔMAP_LRM_ before volume expansion in responders and non-responders are presented in Figure 2B.

**Figure 3:**
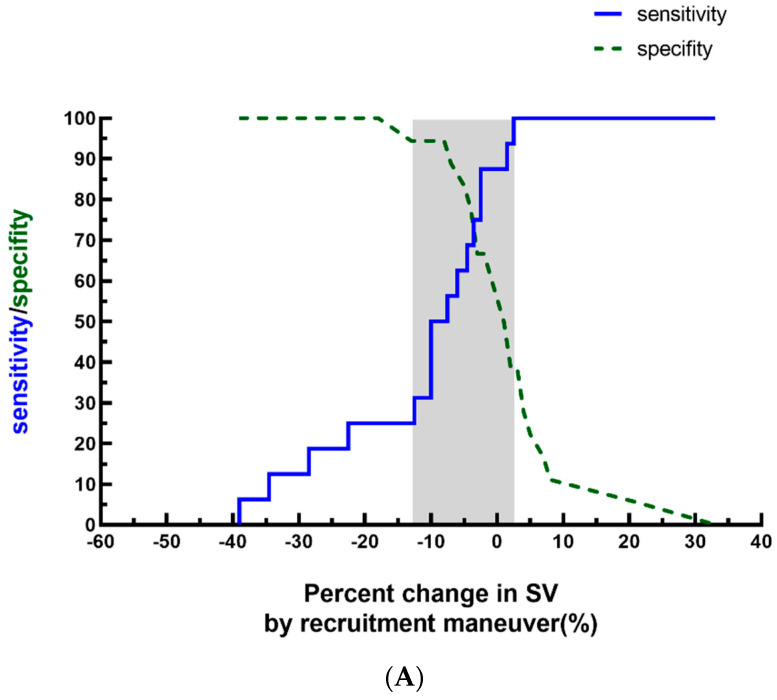

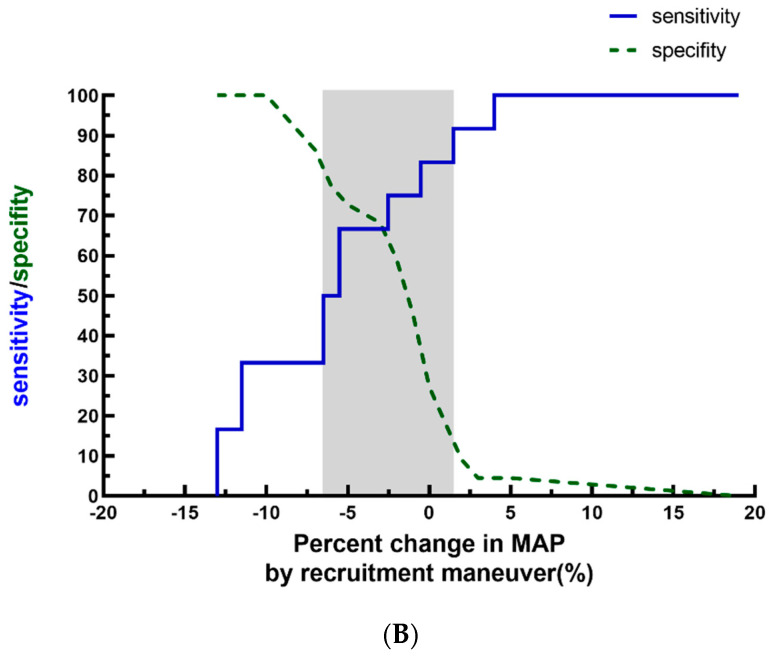
Gray zone of ΔSV_LRM_ (**A**) and ΔMAP_LRM_ (**B**). The blue curve indicates sensitivity, and the green curve indicates specificity. Abbreviations: ΔMAP_LRM_, percent decrease in mean arterial pressure by recruitment maneuver; ΔSV_LRM_, percent decrease in stroke volume by recruitment maneuver; LRM, lung recruitment maneuver, continuous inspiratory pressure: 25 cmH_2_O for 20 s; MAP, mean arterial pressure; and SV, stroke volume.

Figure 3A,B describe the gray zone approach for the ΔSV_LRM_ and ΔMAP_LRM_, and the inconclusive range for SV-fluid responders (ΔSV_LRM_) and MAP-fluid responders (ΔMAP_LRM_) was −13% to 2% and −7% to 2%, respectively.

## 4. Discussion

Our study has demonstrated that the LRM-induced changes of SV could predict fluid responsiveness by discriminating responders from non-responders after fluid loading with an acceptable sensitivity but relatively low specificity. By contrast, the changes of MAP induced by LRM, as well SVV and PPV failed to detect the patients’ fluid responsiveness in children during strategized lung-protection ventilation and OLV. The outcome measures in this study are important and practically useful especially for cardiac anesthesiologists who provide cares to those patients on a daily basis.

Static indicators of cardiac preload have been used for many years to predict the patients’ responses to fluid challenges. A few of the dynamic preload parameters on the ground of cardiopulmonary interactions in mechanically ventilated patients, such as SVV and PPV have been confirmed to be superior to static indices in discrimination of fluid responsiveness of patients in clinical settings. OLV is the most commonly used ventilation technique in cardio-thoracic surgical procedures. Early and timely determination of patients’ fluid status during OLV is crucial to prevent the under- or overloading of fluid. However, conflicting results have existed among the investigators about the predictability of fluid responsiveness by using dynamic indices in adult patients during OLV. Suehiro et al. demonstrated that SVV has shown excellent predictive power in patients undergoing OLV when a tidal volume of 8 mL/kg was delivered to the ventilated lung, but its predictability was compromised when the tidal volume was lowered to 6 mL/kg [15]. Recent research also indicated that PPV failed to predict fluid responsiveness, and SVV had a limited accuracy of 74% to discriminate fluid responders when the ventilated lung received a tidal volume of 6 mL/kg with PEEP at 5 cm H_2_O during OLV in children undergoing video-assisted thoracoscopic surgery [2].

As an essential part of lung protection strategy, low tidal volume has been routinely used intraoperatively in OLV patients to achieve a better outcome. During OLV, cyclic changes of intrathoracic pressure are absent in the non-ventilated lung, while the variations of intrapulmonary pressures are diminished in the ventilated lung with small TV (e.g., 6 mL/kg) and thus the cyclic changes in SVV or PPV were reduced [12,16]. LRM is another important component of lung protective ventilation strategies, and it increases intrathoracic pressure, impedes venous return and increases pulmonary vascular resistance. As a result, the right and left heart preload are decreased, and the right heart afterload is increased, which induces a status of hypovolemia caused by a decrease in right ventricular SV [16]. This physiological mechanism of LRM will increase the predictability of patients’ fluid response and it acts as a new means to evaluate the volume status during OLV. Previous research showed that SV change induced by an LRM was a good discriminator in adults with lung protective ventilation during surgery [9]. And Kimura et al. demonstrated that both a decrease in SV and BP after LRM predicted fluid responsiveness in adult patients during OLV [10]. The results of our study showed that the LRM-induced SV changes could predict fluid responsiveness effectively in pediatric patients during OLV, which were in line with the results of Kimura et al.’s study. And we found that decreased MAP induced by LRM had lower predictability of fluid responsiveness during OLV, which is inconsistent with the above-mentioned studies in adult patients [10]. This disagreement in study results could be attributed to differences in physiology between children and adults, which lie in chest wall compliance, heart rate, mean arterial pressure, arterial vasomotor tone, and aortic elastance. Previous experimental research has indicated that arterial BP response also depends on the arterial tone during volume expansion, which makes it less sensitive to predict fluid responsiveness [17]. Our results in children support those findings. Besides, our study indicated that LRM did not improve the ability of SVV and PPV to predict the fluid responsiveness, which was consistent with the results of previous reports in adults [10]. There was a slight decrease in blood pressure of T3 (before volume expansion) compared with that of T1 (baseline) but the difference was not significant. It may be attributed to the effect of anesthesia medications or hemodynamic changes induced by LRM.

The best area under the curve for SV induced by LRM in our study had a cut-off value of −2% to allow discrimination of children who will benefit from a volume expansion from those who will not, while Kimura et al. and Watanabe et al. have reported that the values of ΔSV_LRM_ threshold were −23.7% and 30% in adult patients undergoing thoracic and spine surgery, respectively [10,18]. The discrepancy of results between theirs and ours could be also ascribed to the difference in study populations such as adults vs. children because Biais et al. have shown that a 30% decrease in SV following LRM could predict fluid responsiveness with a higher sensitivity (88%) and specificity (92%) in patients older than 18 years during neurosurgery [11].

Moreover, our results demonstrated that 61.76% of the patients were in the gray zones for ΔSV_LRM_, and it was significantly lower than the patients for ΔMAP_LRM_ (82.35%), which indicated that ΔSV_LRM_ was a better predictor of fluid responsiveness than ΔMAP_LRM_. However, the ranges of the gray zones for ΔSV_LRM_ and ΔMAP_LRM_ in our study were higher than those in another report (43% and 53%) [10], and by considering the discrepancy of the gray zones among studies, the prudence should be taken in the clinical application of those predictors.

The predictability of fluid responsiveness by the dynamic parameters has been studied in children with CHD, either before or after curative surgery, and either in simple cases (such as ASD or VSD) or complicated ones (such as tetralogy of Fallot) based on the specific pathologies [19,20]. Recently, Kim and colleagues found that LRM enhances the ability of dynamic parameters to predict fluid responsiveness by mimicking a hypovolaemic condition [5]. But scant data are available about assessing the predictability when LRM is applied in pediatric patients undergoing heart surgery during OLV. Children in our study had diagnosis of either ASD or VSD with the existence of an intracardiac shunt. We did not track down data about atrial and/or ventricular compliance, and the changes of intracardiac shunt under the increased intrathoracic pressure induced by LRM. Further investigations are needed to gather more information to address those specific questions.

Several limitations remain in this work. First, the study was conducted in children with CHD during OLV prior to surgical incision and on the left lateral position. LRM could worsen hemodynamic status in those vulnerable patients. Our results may not be extrapolated to critically ill infants or patients with complex congenital heart diseases. Second, our study was conducted under closed chest conditions and the result may be different in those during open chest conditions while the pressure generated by the ventilated lung is transmitted to the atmosphere to some extent. What is more, the variation in thoraco-pulmonary compliance with age is significant. The sample size would be expanded in future to perform age-stratified analyses. Lastly, the observational design had well-known methodologic limits and the sample size was calculated based on a difference in AUC and was not designed for subsequent subgroups [21].

## 5. Conclusions

In conclusion, our preliminary results suggest that the SV changes induced by LRM may have the potential to predict fluid responsiveness in children ventilated with lung-protective settings during OLV. However, further extensive studies are needed to confirm and support this finding.

## Figures and Tables

**Table 1:** Subjects and surgical characteristics. Values are expressed as median (interquartile range (IQR)) or actual numbers (%).

Variables	Overall Population (n = 34)	SV		*p*-Value	MAP		*p*-Value
		Responders (n = 16)	Non-Responders (n = 18)		Responders (n = 12)	Non-Responders (n = 22)	
Age (yr.)	3.3 (2.3–4.3)	2.6 (2.3–4.9)	3.5 (2.1–4.3)	0.746	2.8 (2.1–4.1)	3.4 (2.3–4.4)	0.631
Gender (M/F)	16/18	7/9	9/9	0.492	5/7	11/11	0.459
Weight (kg)	15.1 (12.7–17)	14.5 (12.5–16.0)	15.8 (13.0–17.3)	0.365	14.5 (12.5–16.8)	15.3 (13–17)	0.683
Height (cm)	97.5 (90–104)	94 (90.3–103.5)	101 (89.8–106.0)	0.484	96 (87–104.3)	98 (91–106)	0.736
Type of surgery, n (%)							
ASD	15 (44.1)	7 (44)	8 (44)	0.620	6 (50)	9 (41)	0.439
VSD	17 (50)	8 (50)	9 (50)	0.634	5 (42)	12 (55)	0.360
ASD and VSD	2 (5.9)	1 (6)	1 (6)	0.727	1 (8)	1 (4)	0.588

**Table 2:** Hemodynamic variables in study patients before and after LRM and VE.

Variables	T1	T2	*p* ^a^	T3	T4	*p* ^b^
HR (bpm)	94.1 ± 12.6	85.6 ± 12.0	<0.001 *	94.4 ± 12.6	85.7 ± 13.0	<0.001 *
SAP (mmHg)	93.3 ± 11.9	85.9 ± 11.6	<0.001 *	88.8 ± 11.2	94.5 ± 13.4	<0.001 *
DAP (mmHg)	50.9 ± 7.7	45.8 ± 5.8	<0.001 *	48.3 ± 5.0	49.0 ± 7.0	0.433
MAP (mmHg)	67.7 ± 8.8	60.3 ± 7.0	<0.001 *	64.0 ± 6.6	64.2 ± 8.8	0.852
SV (ml)	12.0 ± 2.9	7.3 ± 2.7	<0.001 *	10.7 ± 2.7	14.0 ± 2.9	<0.001 *
SVV (%)	5.7 ± 2.0			8.2 ± 2.5	3.6 ± 1.3	<0.001 *
PPV (%)	8.1 ± 2.4			6.6 ± 2.1	6.9 ± 2.1	0.451
ΔSV_LRM_ (%)		−7.6 ± 12.3				
ΔMAP_LRM_ (%)		−4.0 ± 7.0				
ΔSV_VE_ (%)					6.3 ± 8.5	
ΔMAP_VE_ (%)					0.1 ± 10.6	

Values are expressed as mean ± SD. SVV and PPV are not generated at T2 due to lack of respiratory variation during LRM and data are not shown in the table. Abbreviations: HR, heart rate; MAP, mean arterial blood pressure; PPV, pulse pressure variation; SVV, stroke volume variation; SD, standard deviation; ΔMAP_LRM_, percent decrease in mean arterial pressure by recruitment maneuver; ΔMAP_VE_, percent change in mean arterial pressure by volume expansion; ΔSV_LRM_, percent decrease in stroke volume by recruitment maneuver; ΔSV_VE_, percent change in stroke volume by volume expansion; LRM, lung recruitment maneuver, continuous inspiratory pressure: 25 cmH_2_O for 20 s; and VE, volume expansion. T1, baseline before lung recruitment maneuver; T2, at the end of lung recruitment maneuver; T3, baseline before volume expansion; and T4, after volume loading. * Statistically significant *p* < 0.05. *p* ^a^: *p* value of T1 vs. T2. *p* ^b^: *p* value of T3 vs. T4.

**Table 3:** Area under the receiver operating characteristics curves for predicting fluid responsiveness of variables before and after LRM.

Variables	AUC	Standard Error ^a^	95% CI	*p*
ΔSV_LRM_ (%)	0.828	0.070	0.660 to 0.935	<0.001
SVV1	0.611	0.100	0.429 to 0.773	0.268
SVV3	0.546	0.105	0.384 to 0.733	0.540
PPV1	0.655	0.099	0.472 to 0.809	0.118
PPV3	0.526	0.107	0.348 to 0.699	0.808

^a^ DeLong et al., 1988 [14]. Abbreviations: ΔSV_LRM_, percent decrease in stroke volume by recruitment maneuver; AUC, area under curve; LRM, lung recruitment maneuver; PPV, pulse pressure variation; SVV, stroke volume variation; SVV1, baseline before lung recruitment maneuver; SVV3, after lung recruitment maneuver; PPV1, baseline before lung recruitment maneuver; PPV3, after lung recruitment maneuver; and CI, confidence interval.

**Table 4:** Area under the receiver operating characteristics curves for predicting fluid responsiveness of variables before and after LRM.

Variables	AUC	Standard Error ^a^	95% CI	*p*
ΔMAP_LRM_ (%)	0.693	0.107	0.512 to 0.839	0.070
SVV1	0.545	0.111	0.366 to 0.716	0.682
SVV3	0.500	0.111	0.324 to 0.676	0.989
PPV1	0.585	0.104	0.404 to 0.751	0.414
PPV3	0.500	0.105	0.324 to 0.676	0.989

^a^ DeLong et al., 1988 [14]. Abbreviations: ΔMAP_LRM_, percent decrease in mean arterial pressures by recruitment maneuver; AUC, area under curve; LRM, lung recruitment maneuver; PPV, pulse pressure variation; SVV, stroke volume variation; SVV1, baseline before lung recruitment maneuver; SVV3, after lung recruitment maneuver; PPV1, baseline before lung recruitment maneuver; PPV3, after lung recruitment maneuver; and CI, confidence interval.

## Data Availability

The data from this study can be obtained from the corresponding author upon request. Due to privacy restrictions, the data are not accessible to the public.

## Abbreviations

ASD: atrial septal defect; AUC: area under the curve; BP: blood pressure; CO: cardiac output; DAP: diastolic arterial pressure; HR: Heart rate; LRM: lung recruitment maneuver; MAP: mean arterial pressure; OLV: one-lung ventilation; PPV: pulse pressure variation; ROC: receiver operating characteristic curve; SAP: systolic arterial pressure; SV: stroke volume; SVV: stroke volume variation; VE: volume expansion; and VSD: ventricular septal defect.
